# The Policy Information Gap and Resettlers’ Well-Being: Evidence from the Anti-Poverty Relocation and Resettlement Program in China

**DOI:** 10.3390/ijerph17082957

**Published:** 2020-04-24

**Authors:** Cong Li, Minglai Li

**Affiliations:** School of Economics and Finance, Xi’an Jiaotong University, Xi’an 710061, China; mllee1996@stu.xjtu.edu.cn

**Keywords:** policy information gap (PIG), subjective well-being (SWB), anti-poverty relocation and settlement program (ARSP)

## Abstract

The widespread dissemination of policy information is necessary for the success of the public policy, but the distribution of information among vulnerable groups has received little attention. We examined a public policy that focuses on the poorest people in China, the anti-poverty relocation and settlement program (ARSP). The infrastructure in the region where the policy is implemented is weak, and the information literacy of resettlers is low. This study analyses the impact of the policy information gap on the subjective well-being of resettlers. We found that the distribution of policy information among the poor is uneven, and the resettlers compare the policy information they obtain with a reference group (working-age people and less-educated people in the same village/community) to generate a policy information gap. The policy information gap indirectly affects subjective well-being by affecting the probability that people will be exposed to risks due to policy. As the policy information gap increases, the subjective well-being of resettlers changes in an inverted U-shape. This impact varies significantly among different groups, policy implementation stages, and resettlement methods. Attention should be paid to the information acquisition ability of the vulnerable groups and the welfare effects of social comparison, and to improve the method of publicizing policy information, which helps to improve the well-being of resettlers.

## 1. Introduction

The anti-poverty relocation and settlement program (ARSP) is a public policy being implemented in China with the dual goals of combating poverty and protecting the ecological environment. In the western region of China, there is much overlap between areas with concentrated poverty and ecologically fragile areas. The government has tried to solve the environmental poverty trap by relocating and resettling residents of these areas [1]. In March 2020, the Chinese government announced that more than 9.2 million people had escaped poverty through ARSP: the incidence of poverty dropped from 10.2% to 0.6%, and the per capita net income of poor households increased from 3416 yuan in 2015 to 9808 yuan in 2019. As an ecological and development policy project, ARSP has relocated farmers to areas closer to towns, industrial parks, tourist attractions and other areas with more convenient production and living conditions and restored the ecology of their previous home regions. Related studies have found that ARSP can bring environmental benefits such as water quality improvement, soil erosion control, and carbon sequestration, which will benefit global stakeholders [1].

Unlike engineering relocation implemented in China in the past (such as the Three Gorges Project Resettlement), ARSP emphasizes the voluntary relocation of farmers [2], although some studies refer to it as “induced voluntarism” or “compulsory voluntarism” [3,4]. To achieve the goal of the voluntary relocation of farmers, the Chinese government engaged in information disclosure during the implementation of the policy, hoping to improve farmers’ understanding of the policy. At the same time, ARSP, as one of the main features of the “precision poverty alleviation” strategy of China, emphasized that the poorest people should be accurately identified as the relocation target. To avoid the phenomenon of rent-seeking and to accurately relocate the poorest people, extensive supervision by the masses is necessary. Democratic supervision objectively requires the widespread dissemination of policy information (including identifying resettlers, homestead retreat, housing subsidy, employment support, industrial development, and social security for the place of relocation, etc.), and the population’s full understanding of that information and corresponding institutional arrangements. For example, local governments in China have adopted measures such as public announcements, dissemination of promotional materials, the convening of villagers ’meetings, and the establishment of villager supervision teams. Besides, ARSP involves a large number of post-relocation assistance policies, such as employment training and industrial support. The full understanding and use of policy information will help resettlers realize their livelihood transformation and escape from the poverty trap. Previous studies have often focused on whether ARSP can improve the income and well-being of resettlers [2,5,6]. However, they have ignored the full dissemination and understanding of policy information as the prerequisite for policy success. Most areas where ARSP is implemented have poor living conditions, severe production conditions and weak infrastructure. The information acquisition capacity of resettlers is insufficient, which is closely related to information poverty [7,8] and leads to an unequal understanding of policies. Therefore, the relationship between policy information in ARSP areas and the well-being of resettlers has high research value.

Satisfaction scores of subjective well-being (SWB) are relatively easy to collect, which offers a practical and easily implementable tool for well-being comparisons and is widely used in the world [9,10]. Previous research has shown that social comparison with reference groups is an important factor affecting SWB [11]. The theory of relative deprivation and relative income found that it is not the absolute value of income but the relative value that truly affects SWB [12,13]. Farmers in ARSP areas have a low level of information literacy, and their understanding of policy information is uneven, which easily creates an information gap. Therefore, when studying the impact of policy information (such as identifying resettlers, homestead retreat, housing subsidy, employment support, industrial development, and social security for the place of relocation, etc.) on SWB, we need to consider social comparisons and pay attention to the distribution of policy information among the population if we want to understand the impact of the policy information gap on SWB. This can help us to better understand who is the key group requiring policy advocacy when implementing public policies, which vulnerable groups should be given extra attention for certain types of information, and which measures can help avoid unnecessary welfare losses.

Therefore, from the perspective of social comparison, we constructed a policy information gap (PIG) indicator. This indicator measures the difference between resettlers’ understanding of ARSP policy information and others. On this basis, we measured the impact of the PIG on SWB and tested its impact mechanism through a mediating effect method. Finally, we analysed the differences in the impact of the PIG on the SWB of different reference groups, populations, and policy environments.

## 2. Literature Review

Much of the previous literature has focused on the relationship between relocation projects and the well-being of resettlers. Some studies show that although relocation improves the housing conditions of resettlers, it worsens their economic, social, and cultural well-being [6,14]. However, some studies have shown that relocation projects help resettlers obtain better public services, increase income, and improve ecosystem services [1,4,5,15]. Some scholars have tried to explain the results of this difference, arguing that the relocation has uncertain effects on the material well-being of the resettlers [16]. The reason, time, and resettlement methods are all important factors affecting the economic well-being of resettlers [2,15].

Since the 1970s [17,18,19,20,21], research on information poverty has gradually emerged. Information poverty refers to a kind of missing or unsatisfied information demand due to improper group information behavior [8,22,23], insufficient information supply [24,25], individual lack of information acquisition ability [7,8], etc. Information-poor groups include farmers [26], the elderly [22], etc. Most of the research sites are located in Africa and developing countries [27,28]. The causes of information poverty among these information-vulnerable groups can be divided into their personal factors (e.g., information capacity [29,30] and information exchange [31]) and environmental factors (e.g., information resources [7], information infrastructure [32,33]). A large number of studies use objective test questions or subjective self-assessments to test the respondents’ degree of information absorption [34,35,36]. According to the knowledge gap hypothesis [37], groups trapped in information poverty are at a disadvantage in terms of information absorption, creating an information gap with other groups, and this gap will grow wider. The information gap and information poverty are closely related to economic poverty [38,39]. Information poverty and economic poverty are mutually causal, and people with information poverty often fall into the cycle of “information poverty–economic poverty–information poverty” [39]. Studies have also focused on the impact of the information gap on parents’ choice of school [40]. Therefore, information gaps and information poverty can lead to economic poverty and inequality and ultimately affect well-being.

Well-being has multiple measures [10]. Commonly used measures include income [5], SWB [41,42], and the United Nations Human Development Index. The reason for this variety is that well-being is a comprehensive concept that involves many aspects, such as living conditions, health, safety, social connections, and physical conditions [43,44]. The related disciplines of well-being include economics, sociology, and psychology [42]. Generally, well-being can be divided into subjective and objective aspects. SWB focuses on people’s emotional experience and satisfaction, and self-reported happiness has received widespread attention from researchers [45]. The influencing factors of SWB include income [46], age [47], gender [48], marriage [49], health [50], education [48], public policy [51], and so on. In addition to the influence of absolute factors, the inequality brought by comparison between groups also affects SWB, such as income inequality [52]. Relative deprivation is an inequality index defined at the individual level [53] that reflects the social comparison between individuals and reference groups. Common reference group selection criteria include geographical location [54], demographic characteristics [55], and social relationships [11]. A large amount of the literature has focused on the effect of relative income on SWB [56,57,58].

It can be seen from the existing literature that information gaps and information poverty can lead to economic poverty and inequality, but research on the well-being of information-poor groups is still scarce. Therefore, we draw on the theory of relative deprivation, pay attention to information distribution among the poor, and study the impact of the policy information gap on SWB.

## 3. Materials and Methods

### 3.1. Research Area and Survey

The data used in this paper is from a rural household livelihood and environment survey conducted by Xi’an Jiaotong University in 2015 in Shaanxi Province, China. Shaanxi Province is located in the western part of China. It has a variety of landforms on the Loess Plateau, the Guanzhong Plain, and the Qinling Mountains. It is the water source of the South-to-North Water Diversion Project in China and has 25 national nature reserves. At the same time, the population of those living in poverty is large, and 56 of the 107 counties in the province are poor counties (2016 data). According to the national “13th Five-Year Plan” for ARSP announced by the Chinese government, Shaanxi is planning to take anti-poverty measures and achieve ecological protection through the relocation and resettlement of 1.25 million people. Shaanxi is one of five provinces with a resettlement population of 1 million or more (the remaining provinces are Guizhou with 1.3 million, Sichuan with 1.16 million, Hubei with 1 million, and Guangxi with 1 million), so the survey data for ARSP in Shaanxi are representative. In Shaanxi Province, the key areas for policy implementation are overlapping areas of ecological fragility and poverty, including the Qinba Mountains, Liupan Mountains, Luliang Mountains, Baiyu Mountains and the Rocky Mountains along the Yellow River. 

**Figure 1:**
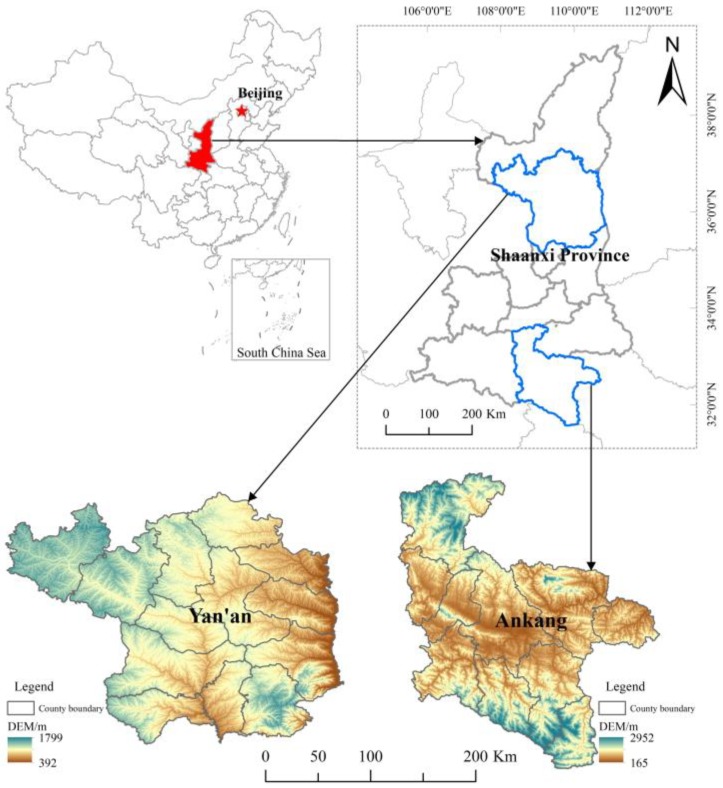
The geographic location of the sample area.

As shown in Figure 1, the sample areas we surveyed included Ningshan County, Hanbin County, Ziyang County in Ankang City, and Wuqi County in Yanan City. They are located in the Qinba Mountains and Baiyu Mountains, and have or are near to nature reserves. They are the key areas for implementing the ARSP. According to the list of administrative villages provided by the Bureau of Statistics, we randomly selected villages or communities involving ARSP. In the investigation of villages and communities, we randomly selected respondents who were at home that day. The survey includes family population information, participation in ARSP, and family livelihood activities. In the investigation, we adopted various methods to ensure data quality. The survey uses a combination of structured questionnaires and semi-structured interviews. All investigators have undergone systematic training. After the questionnaires were collected, they were reviewed by supervisors. Questionnaire data were checked for logic after they were entered. Respondents were fully informed and consented to data collection before the survey. In the entry and use of data, we fully paid attention to protecting the information of the respondents. This survey received a total of 953 valid questionnaires, of which 550 were relocated households, accounting for 57.71%. In this study, we used the sample of 550 relocated households.

### 3.2. Dependent Variable

SWB is a common indicator of well-being [9,10,41,42], which can reflect the life satisfaction of respondents and is easy to observe. We used “your overall evaluation of your life last year” to obtain the SWB data of the resettlers. The resettlers answered this question by considering their life satisfaction. The options include “very satisfied,” “satisfied,” “general,” “not satisfied” and “very dissatisfied,” which are ranked 1–5, respectively. In the further processing of the data, we adjusted it to a positive index, in which 1 means “very unhappy” and 5 means “very happy.” The sample average was 3.516, indicating that the life satisfaction of most of the respondents was between “general” and “happy.”

### 3.3. Independent Variables

We asked the respondent “Do you know the ARSP policy?” to obtain the resettlers’ understanding of the policy information. Respondents gave a score of 1–5 based on their understanding of the policy information. Before asking this question, we introduced the information included in the ARSP policy to the resettlers: identifying resettlers, homestead retreat, housing subsidy, employment support, industrial development, and social security for the place of relocation, etc. With reference to related research on relative income [12,59,60], the policy information gap (PIG) measures the difference between the respondent’s policy understanding and the average policy understanding of the reference group and is calculated from the logarithmic difference between the two. Because we were concerned about the gap between the respondent and the reference group, we calculated the absolute value of the calculation results, which helped us focus on the analysis of policy information inequality. The final formula for calculating the PIG is shown below.

(1) $$PIG_{i}=\left|\ln\frac{policy_{i}}{peer_{i}}\right|=\left|\ln policy_{i}-\ln peer_{i}\right|$$

The division of the reference group is essential for the calculation of the PIG, and the basis of the division includes geography, education level, and age [54,55]. We have comprehensively considered the above division methods and gradually introduced age and education level based on geographical factors (residents living in the same village or community as the respondent) to more accurately identify the reference that affects the SWB of the resettlers. To verify the validity of selecting the population that lives in the same village or community as the reference group among geographical factors, we performed a placebo test by comparing the population of the same county but different villages or communities with the respondent. According to the above calculation method, we defined six types of policy information gaps. (1) PIG is the absolute value of the difference between the logarithm of the respondents’ understanding of policy information and the logarithm of the average policy understanding of residents in the same village or community. (2) PIG-Placebo is the absolute value of the difference between the logarithm of the respondents’ understanding of policy information and the logarithm of the average policy understanding of residents in the same county but in different villages or communities. (3) PIG-elderly is the absolute value of the difference between the logarithm of the respondents’ understanding of policy information and the logarithm of the average policy understanding of the elderly residents in the same village or community. (4) PIG-working age is the absolute value of the difference between the logarithm of the respondents’ understanding of policy information and the logarithm of the average policy understanding of the working-age residents in the same village or community. (5) PIG-high educated is the absolute value of the difference between the logarithm of the respondents’ understanding of policy information and the logarithm of the average policy understanding of the residents with high education levels in the same village or community. (6) PIG-low educated is the absolute value of the difference between the logarithm of the respondents’ understanding of policy information and the logarithm of the average policy understanding of the residents with low education levels in the same village or community.

We controlled for personal and family factors that may affect SWB, including age (respondent’s age in years), gender (1 = male, 0 = female), marital status (1 = unmarried; 2 = married; 3 = divorced or widowed), health (1 = bad; 2 = average; 3 = good), education (respondents’ years of education), labour population proportion (proportion of the working-age population aged 15 to 64 in the family), and income (the logarithm of household income per capita). Referring to previous studies on the effect of age on SWB [47,61], we also introduced the square of age. At the same time, we controlled for differences in the implementation of the relocation and resettlement policies, including resettlement methods after the relocation (1 = centralized resettlement; 0 = decentralized resettlement), types of resettlement (1 = poverty alleviation; 2 = ecological restoration; 3 = engineering project-induced; 4 = disaster-related; 5 = other) and geographic regions (1 = Ankang; 0 = Yan’an). In addition, in order to analyse the mechanism of PIG affecting SWB, we introduced two variables: loss (whether the family’s economic interests, emotions, and social relations have deteriorated or suffered losses since the relocation. 1 = yes; 0 = no) and social network (the logarithm of family members’ communication costs last month). The descriptive statistics of all the variables selected are shown in Table 1.

### 3.4. Statistical Analysis

Since SWB is an ordered discrete variable, the traditional OLS regression method was not suitable for this data type. We chose the ordered probit model to analyse the effect of PIG on SWB, as shown below:

(2) $$SWB_{i}=\beta_{0}+\beta_{1}PIG_{i}+\beta X_{i}+\mu$$

(3) $$SWB_{i}=\{\begin{array}{l} 1,\;SWB_{i}^{*}\leq r_{1}\\ 2,r_{1}<SWB_{i}^{*}\leq r_{2}\\ 3,r_{2}<SWB_{i}^{*}\leq r_{3}\\ 4,r_{3}<SWB_{i}^{*}\leq r_{4}\\ 5,r_{4}<SWB_{i}^{*} \end{array}$$

Among them, $SWB_{i}$ represents the subjective well-being of the resettler *i*, $SWB_{i}^{*}$ represents the latent variable of SWB, $PIG_{i}$ represents the policy information gap of resettler *i*, and $X_{i}$ represents other independent variables. Cut-off points are $r_{1}$–$r_{4}$, which divide the SWB of resettlers into different levels. When the random disturbance term μ follows a normal distribution, the maximum likelihood estimator of the model can be obtained. 

Based on this regression, we further performed the following: (1) A placebo test was performed on samples from the same county but different villages or communities as the respondent, which was used to test the effectiveness of the reference group; (2) the reason for the inverted U-shaped relationship between PIG and SWB was analysed using the mediation effect model; (3) the age and education level in the reference group were further subdivided to more accurately identify the reference groups; (4) respondents were grouped and regressed according to gender, age, and education level in order to identify groups sensitive to the PIG; (5) different policy scenarios (policy implementation stages and resettlement methods) were examined to analyse the policy approach to reduce welfare losses; (6) all regressions reported the standard error of robustness to solve the problem of heteroscedasticity.

## 4. Results

### 4.1. Policy Information Gap and Subjective Well-Being: Phenomenon and Mechanism

Table 2 shows the regression results and mechanism analysis between SWB and the PIG. Column (1) reports the regression results for SWB and the PIG calculated using samples located in the same village (community) as the reference group; column (2) serves as the control group of column (1) and reports the results of a placebo test, where the PIG is calculated using samples from the same county but different villages (communities); columns (3), (4), and (5) analyse the influence mechanism of the PIG on SWB and examine the intermediary effects of losses suffered due to relocation.

**Figure 2:**
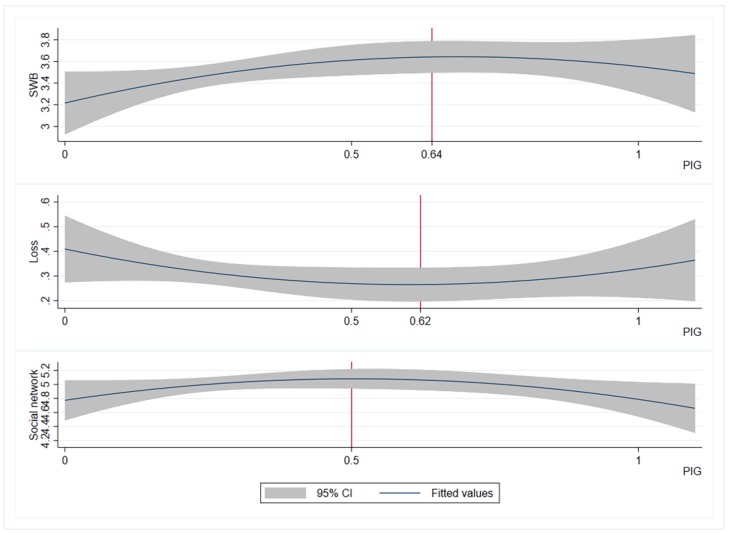
The fitting curve of subjective well-being (SWB), loss and social network with policy information gap (PIG).

The coefficient of the PIG in column (1) is significantly positive, and the coefficient of the square term of the PIG is significantly negative, indicating that the SWB of the resettlers first increases and then decreases with the increase in the PIG, showing an inverted U-shaped relationship. As shown in Figure 2, we plotted the curve of SWB versus PIG. We believe that the shape of the curve is related to the probability of loss due to ARSP and the sharing of risk by the social network, which is verified in the regression results in columns (3), (4), (5). The quadratic term of age is significantly positive, indicating that there is a U-shaped relationship between SWB and age, which is consistent with existing studies [47,61]. We calculated the inflection point as 40.3 years old based on the coefficient of age and the quadratic term of age, which means that before 40.3 years old, the SWB decreases with age, and after 40.3 years old, the SWB increases with age. The coefficient of health is significantly positive, indicating that when other factors are unchanged, the probability of obtaining a higher level of SWB will increase with the improvement of health. At the same time, the reason for the migration also has a significant impact on the SWB of the resettlers. Taking poverty-alleviation resettlers as a reference, the probability of project resettlers obtaining a high level of SWB is significantly lower because project resettlement is often accompanied by involuntary relocation and resettlement.

The PIG in column (2) was calculated from samples located in the same county but different villages (communities) as the respondent and named PIG-Placebo. The regression results show that the significance of PIG-Placebo is significantly lower than that of PIG in column (1), and it does not significantly affect the resettlers’ SWB. This shows that it is not appropriate to use different villages (communities) in the same county as the reference group; it is instead appropriate and effective to select samples in column (1) that live in the same village or community as the reference group. This result is in line with our intuition and expectations. The sample area is located in the western mountainous areas of China. Many of the villages and communities where the respondents lived were located in the mountain hinterland or at the foot of the mountain, and the transportation was inconvenient. Except for the core area of the county seat, residents in the same county-level administrative area are scattered. The natural barriers in the mountains make it difficult for resettlers to interact with other people outside the village (community).

We used column (3), (4), (5) to analyse the influence mechanism of PIG on SWB, that is, the PIG indirectly affects SWB by affecting the probability of loss due to relocation. As shown in Figure 2, when PIG increases from 0 to 0.5, the frequency of social network interaction of resettlers increases and the probability of loss decreases, and SWB increases. However, when the PIG exceeds 0.5, the frequency of social network interaction of the resettlers decreases; when it exceeds 0.62, the probability of loss increases; when it exceeds 0.64, the SWB decreases. Social networks are a natural way for resettlers to share risks. The frequency of social network interactions significantly affects the resettler’s loss probability, and the loss probability significantly affects the resettler’s SWB. Therefore, as the PIG gradually increases, the social network interaction frequency changes, then the probability of loss changes and eventually the SWB changes.

### 4.2. Comparisons: Reference Groups for Policy Information

Table 3 reports the regression results from further subdividing the reference group based on living in the same village (community). Columns (6) and (7) show the impact of reference groups of different ages on SWB. Columns (8) and (9) show the impact of reference groups with different educational levels on SWB.

In column (6), the recalculation of the PIG and its quadratic term using middle-aged and older people in the same village (community) as the reference group has no significant effect on SWB. However, in column (7), the recalculated PIG and its quadratic term using working-age people of the same village (community) as the reference group does have a significant effect on SWB. When other factors remain unchanged, the SWB of the resettlers first rises and then decreases with the increase in the PIG of working-age people in the same village (community), showing an inverted U-shaped relationship, which is consistent with the results obtained in column (1). The results of columns (6) and (7) indicate that when comparing policy information, resettlers are mainly based on neighbouring working-age people, not the elderly in the same village (community). In addition, we paid attention to the effect of age on SWB when introducing different age reference groups. Consistent with the results in column (1), the quadratic terms for age in column (6),(7) are significantly positive and age is significantly negative, which indicates that SWB is U-shaped with age. In columns (6) and (7), the U-shaped inflection points are at 42.1 and 43.1 years old, which is similar to the result of column (1).

In column (8), the recalculated PIG and its quadratic terms, using residents with a low education level in the same village (community) as a reference group, have a significant impact on SWB. When other factors are unchanged, the SWB of the resettlers follows the PIG of residents with a low education level in the same village (community), first rising and then declining, showing an inverted U-shaped change which is consistent with the results obtained in columns (1) and (7). However, in column (9), the PIG and its quadratic terms calculated using residents with high education levels in the same village (community) as a reference group have no significant effect on SWB. Combining the results of columns (8) and (9), resettlers tend to choose neighbouring residents with lower education levels for the social comparison of policy information rather than more educated groups.

### 4.3. Groups Sensitive to the Policy Information Gap

Table 4 reports the results of the group regression. We classified the respondents according to gender, age, and education level and studied the sensitivity of different groups to the PIG. Among them, columns (10) and (11) respectively show the impact of PIG on SWB among male and female respondents; columns (12) and (13) respectively show the impact of PIG on SWB among working-age and older people; columns (14) and (15) respectively show the impact of PIG on SWB among highly educated and less-educated people.

Column (10) used male samples to test the effect of PIG on SWB. The results show that the inverse U-shaped effect of PIG on SWB in male samples is still significant, but the effect of using female samples in column (11) is no longer significant. Column (12) shows that the effect of PIG on SWB in the sample of the working-age population is not significant, but the effect of PIG on SWB in the sample of older people in column (13) is significant, showing an inverted U-shaped effect. Column (14) shows that the impact of PIG on SWB in the samples with high education levels is not significant, but the effect of PIG on SWB in samples with low education levels in column (15) is significant, showing an inverted U-shaped influence.

### 4.4. Policy Environment: Catalyst for Policy Information Gap

Table 5 shows the difference in the impact of the PIG on SWB in different policy environments. Columns (16) and (17) compare the policy pilot phase (relocation before 2011) and the large-scale policy implementation phase (relocation time after 2011). Columns (18) and (19) compare the differences between two typical resettlement models, namely, centralized resettlement and decentralized resettlement.

Column (16) shows the inverted U-shaped effect of the PIG on SWB in the policy pilot phase, and this effect was not significant during the large-scale implementation phase of the policy in column (17). Column (18) shows that the effect of the PIG on SWB under the centralized resettlement model is very low, while column (19) shows that the effect of the PIG on SWB under the decentralized resettlement model has a significant U-shaped effect. This result is consistent with our expectations. Under the situation of policy pilot stage and decentralized resettlement, the social network of resettlers has been damaged and the risk cannot be effectively shared. Therefore, the impact of PIG on SWB is more significant.

## 5. Discussion

Previous studies on social comparison and well-being have focused on a wide range of issues, including income [62], health [63], living standards [64], academic performance [65], and so on. However, for resettlers participating in ARSP policies, evenly distributed, accessible and understandable policy information is very important. Policy information is related to whether they decide to participate in the project and how to make a livelihood transformation after participating in the project. Therefore, resettlers have sufficient motivation to pay attention to policy information and make social comparisons with neighborhoods. However, the previous research on information did not pay attention to this point, and most of them focused on information acquisition methods and the consequences of insufficient information [38,39]. To overcome the shortcomings of previous studies, we draw on the theory of relative deprivation, pay attention to information distribution among the poor, and study the impact of the policy information gap on SWB. Therefore, our research incorporates policy information into the category of social comparison and analyses the mechanism of impact on SWB through social networks and loss probabilities, enriching research on social comparison and well-being. 

Most of China’s rural areas are familial settlements, with many relatives in the same village (community) and intensive neighbourhood exchanges, forming a natural social network of shared benefits and risks [66,67]. Individuals in social networks are highly similar [68], and people tend to communicate with people who are similar to themselves. According to reciprocal altruism [69,70], the premise of altruistic behavior is to expect higher returns than costs. We can explain the reasons for the inverted U shape of social networks and PIG based on the above two theories. When the PIG is less than 0.5, the difference between the resettlement group and the peer group is small, and the similarity is higher. At the same time, the resettler expects others to have a higher probability of giving back information of the same value, because everyone has a similar level of policy information when the PIG is low. Therefore, the frequency of social interaction will increase with the increase of PIG. However, when the PIG exceeds 0.5, the difference between the resettlers and the peer group in terms of policy information increases, and the degree of similarity decreases. At this time, the policy information is unevenly distributed, the policy information held by the resettlers is very polarized, and the probability of expecting others to return the same value is reduced. As a result, the frequency of social interaction of resettlers has decreased. Considering that social networks are the main way for resettlers to share risks [67], changes in social frequency will affect the probability of risk. When the PIG is low, the social network fully disseminates information, which helps resettlers share risks and improve SWB. However, when PIG is high, the level of inequality intensifies, and at the same time social network support weakens, resettlers are unable to use policy information for livelihood transformation and the probability of loss due to resettlement increases, thereby reducing SWB.

The difference in the impact of PIG on SWB between different age groups is related to the phenomenon of “hollowing” [71] in rural areas of China. In the rural areas of China, working-age people mostly choose to work outside the home (78.32% in our survey), and the elderly and children are at home [72]. Therefore, although the working-age population bears the burden of generating income and ensuring a stable livelihood transition for the family, those who receive policy information directly at home are mostly elderly. Information acquisition plays a very important role in family decision-making [73]. Especially when faced with the exogenous shocks of relocation and resettlement, it is essential for a family that the working-age members have sufficient policy information and can make full use of the information to achieve a smooth transition in family livelihood. In Chinese family pensions, children support the elderly, and the elderly also provide intergenerational support for their children [74]. Therefore, although these elderly groups withdrew from the labour market and are supported by their children, they were more sensitive to policy information related to the core interests of the family. At the same time, older people in China tend to live at home [75]. In our survey, we found that the resettlement communities are equipped with excellent cultural and sports facilities, which provide a convenient place for older people at home to communicate with each other. However, their ability to understand policy information is limited [76,77], and they no longer play a significant role in family production and life decisions. For social comparisons of the policy information held by resettlers, they tend to choose the working-age population in other families as a reference to support family decision-making.

The reason for the difference in the impact of PIG on SWB between different education groups is that highly educated people have an information advantage: there are more extensive channels (such as using the Internet) through which they can obtain and identify policy information, and they have a better understanding of that information [78]. Resettlers tend to think that it is reasonable and natural for highly educated groups to be superior to themselves in understanding policy information, which will form an anchoring effect [79]. Only when less-educated groups that do not have an information advantage exceed themselves in policy information will they have a psychological impact and affect SWB. Therefore, resettlers tend to use less educated residents who do not have information advantages as a reference group for policy information. Besides, policy information has different marginal effects on groups with higher education levels than on those with lower education levels. According to the law of diminishing marginal returns [80], as input increases the marginal effect shows a downward trend. Diminishing marginal returns have been widely used in economic society, including campaigns [81], research and development [82], education [83,84], and happiness [85]. Therefore, highly educated groups have apparent advantages in the acquisition and understanding of policy information, and with higher storage of information and a lower marginal impact of policy information, less-educated groups occupy a disadvantaged position in information and increased difficulty in obtaining and understanding policy information. As the less-educated groups have less policy information, and they may misread the information they have, so it will be more likely to cause information inequality and asymmetry, and the marginal impact of policy information will be higher. Therefore, less-educated groups are more sensitive to the PIG.

Moreover, the impact of PIG on SWB is also various in different genders. We believe that the reason for this phenomenon is gender inequality in rural areas of China [86], where men have higher decision-making power. This is consistent with the higher proportion of male heads of household in the sample (92.16%). As a head of the household, men need to organize family members to make a livelihood transition in time to deal with the risks that resettlement may bring. They are more sensitive to policy information and other factors that may affect the family’s future livelihood strategies. Therefore, the impact of PIG on SWB in males is significant. Although the Chinese government advocates equality between men and women, the concept of “boy preference” still exists in rural areas of China [87]. Women are more responsible for housework and child care. In addition, women in rural areas in China have lower educational opportunities than men [87]. In our survey, the average length of education of female respondents was 5.1 years, while that of male respondents was 6.8 years. Lower education levels also reduce women’s ability to understand policy information [78]. Therefore, women pay less attention and sensitivity to the information gap.

Finally, it must be noted that the impact of PIG on SWB is more pronounced during the policy pilot phase and non-centralized resettlement, which is consistent with previous research focusing on the policy environment on the well-being of resettlers [15]. The Chinese government tends to conduct pilot projects in some regions before implementing policies on a large scale [88]. During the pilot phase of the policy, because a small number of farmers are included in the ARSP, the policy information is unevenly distributed so it is easier to generate a social comparison of the policy information that affected the SWB of the resettlers. At the large-scale implementation stage of the policy, eligible farmers are widely included in the relocation and resettlement plan, the social network of the resettlers widely spreads the policy information, the policy information is more evenly distributed, and the policy information is less unequal. Therefore, the probability that the social comparison of policy information affects SWB is low. The impact of the PIG on SWB in the two resettlement modes is related to the role of the resettlement social network in spreading information. In the concentrated resettlement areas, the resettlers gather and live, and the flow of information about relocation and resettlement is smoother [89]. Under the non-centralized resettlement model, resettlers are scattered into the communities of local residents, and it is challenging to obtain policy information from social networks. When policy information spreads widely with the community’s social network, the degree of inequality in the distribution of policy information decreases and the impact of the PIG on SWB decreases.

Our research focuses on the population in China’s poorest regions, and strictly analysed the impact of policy information distribution on well-being at the community level. This paper establishes a link between social comparison, policy information and well-being, and provides a new perspective for the analysis and evaluation of public policies. This study helps policymakers to more effectively promote policy and give more care to information-sensitive people (for example, give them additional policy explanations), reduce welfare losses, and improve the well-being of policy audiences. The main limitation of this research is that our research uses survey data from Shaanxi, China, and related research conclusions may not be generalizable in other countries. Since ARSP in other regions of China has the same goals and practices, our research conclusions still have the value of promotion and reference. In addition, we focus on anti-poverty and ecological resettlers. Whether the research conclusions apply to other types of migrants needs further study.

## 6. Conclusions

We used micro data from Shaanxi, China to analyse the impact of PIG on the well-being of resettlers. The results of the study indicate that SWB changes with the PIG in an inverted U shape. The imbalance in the distribution of policy information will affect the frequency of resettlers’ social network interaction. Social network is related to the risk sharing of relocation and ultimately affects the well-being of resettlers. The resettlers will compare the policy information they have obtained with the neighbors of the same village or community, especially with the working-age population and low-educated resettlers of the same village or community. Among different populations, male, elderly, and low-educated resettlers are more sensitive to PIG. In different policy environments, resettlers in the pilot phase and decentralized resettlement are more sensitive to PIG.

By analyzing the mechanism by which PIG affects well-being at the resettler level, our research complements the study of the social comparative behaviour of information and well-being and is instructive for the formulation of public policies and publicity measures. It also provides different perspectives on the well-being of the poor. Taking China’s ARSP as an example, improving the information acquisition capacity of resettlers is more valuable than publishing policy information on bulletin boards. At the same time, additional attention should be given to information-disadvantaged groups such as the elderly, and less-educated groups through initiatives such as popularizing policy in a way that is easy for these groups to understand and to prevent them from being disadvantaged by the policy information. Besides, policymakers must correctly understand the welfare effect of social comparison, pay attention to the state of policy information among of the reference group, and strive to maximize social well-being while not harming the interests of the reference group. Finally, attention should be paid to the method of disseminating policy information through the resettlers’ social network. During the pilot phase of the policy, the policy should be implemented in a specific area, and the target groups should be concentrated. These measures can widely disseminate policy information through social networks and reduce the gap in understanding policy information.

## Figures and Tables

**Table 1:** Descriptive statistics of variables.

Variable	Mean	Std.Dev.	Min	Max
Subjective well-being (SWB)	3.516	0.995	1	5
PIG	0.420	0.275	0	1.099
PIG-Placebo	0.428	0.278	0	1.036
PIG-elderly	0.404	0.311	0	1.386
PIG-working age	0.403	0.267	0	1.099
PIG-high educated	0.426	0.305	0	1.447
PIG-low educated	0.399	0.283	0	1.386
Age	51.24	12.51	21	87
Gender	0.617	0.486	0	1
Marital status	2.071	0.392	1	3
Health	2.243	0.831	1	3
Education	6.184	3.893	1	16
Proportion of working-age members	0.751	0.222	0	1
Income	8.782	1.249	1.204	11.71
Type of relocation	0.729	0.445	0	1
Reason for relocation	3.247	1.466	1	5
Loss	0.316	0.465	0	1
Social network	4.983	0.962	1.253	8.517
Area	0.835	0.372	0	1

**Table 2:** Ordered probit regression and intermediary effect analysis of subjective well-being on policy information gap.

Variables	(1)	(2)	(3)	(4)	(5)
	SWB	SWB	Loss	Social Network	SWB
Loss (reference to no)					−0.399 ***
					(0.113)
PIG	1.825 **		−2.328 **	1.589 **	1.568 **
	(0.733)		(1.007)	(0.677)	(0.731)
PIG squared	−1.410 **		1.872 **	−1.593 ***	−1.209 *
	(0.701)		(0.916)	(0.602)	(0.706)
PIG-Placebo		1.532 *			
		(0.786)			
PIG-Placebo squared		−1.040			
		(0.736)			
Age	−0.0479 *	−0.0463 *	−0.00426	−0.00148	−0.0589 **
	(0.0283)	(0.0277)	(0.00611)	(0.00419)	(0.0288)
Age squared	0.000592 **	0.000579 **			0.000697 **
	(0.000278)	(0.000273)			(0.000283)
Proportion of working-age members	0.754 ***	0.675 ***	0.122	0.243	0.812 ***
	(0.263)	(0.252)	(0.295)	(0.198)	(0.265)
Income	0.0535	0.0442	−0.0560	0.0319	0.0461
	(0.0413)	(0.0387)	(0.0519)	(0.0394)	(0.0422)
Education	0.0182	0.0220	−0.00155	0.0372 **	0.0180
	(0.0178)	(0.0173)	(0.0207)	(0.0148)	(0.0179)
Health	0.256 ***	0.263 ***	−0.267 ***	0.107 *	0.224 ***
	(0.0688)	(0.0676)	(0.0823)	(0.0602)	(0.0700)
Marital status (reference to unmarried)					
Married	−0.141	−0.182	0.0802	0.152	−0.115
	(0.281)	(0.285)	(0.295)	(0.224)	(0.277)
Divorced or widowed	−0.311	−0.359	0.159	−0.0889	−0.286
	(0.310)	(0.314)	(0.353)	(0.265)	(0.306)
Gender (reference to female)	−0.00183	−0.0270	0.164	−0.0697	0.0195
	(0.111)	(0.109)	(0.145)	(0.106)	(0.112)
Type of relocation (reference to decentralized resettlement)	−0.00190	0.00784	0.0592	0.0130	0.00456
	(0.120)	(0.116)	(0.159)	(0.113)	(0.120)
Reason for relocation (reference to poverty alleviation)					
Ecological restoration	0.216	0.113	0.180	0.162	0.229
	(0.197)	(0.188)	(0.260)	(0.186)	(0.199)
Project-induced	−0.460 **	−0.521 **	0.673 **	−0.342 *	−0.389 *
	(0.226)	(0.223)	(0.285)	(0.180)	(0.225)
Disaster-related	−0.118	−0.162	0.485 ***	−0.0804	−0.0639
	(0.130)	(0.126)	(0.173)	(0.112)	(0.130)
Other	−0.134	−0.167	0.801 ***	−0.101	−0.0360
	(0.147)	(0.143)	(0.217)	(0.142)	(0.151)
Area (reference to Yan’an)	0.0583	−0.0271	−0.656 ***	−0.116	−0.0328
	(0.190)	(0.155)	(0.195)	(0.139)	(0.191)
cut1	−0.701	−0.937			−1.258
	(0.840)	(0.813)			(0.869)
cut2	0.0118	−0.236			−0.540
	(0.836)	(0.808)			(0.862)
cut3	0.946	0.695			0.412
	(0.839)	(0.812)			(0.862)
cut4	2.275 ***	2.020 **			1.762 **
	(0.844)	(0.816)			(0.864)
Constant			1.099	3.946 ***	
			(0.808)	(0.520)	
Observations	477	503	477	471	477

Robust standard errors in parentheses, *** *p* < 0.01, ** *p* < 0.05, * *p* < 0.1.

**Table 3:** Reference groups for policy information.

Variables	(6)	(7)	(8)	(9)
	SWB	SWB	SWB	SWB
PIG-elderly	−0.268			
	(0.499)			
PIG-elderly squared	0.270			
	(0.464)			
PIG-working age		1.627 **		
		(0.746)		
PIG-working age squared		−1.222 *		
		(0.727)		
PIG-low educated			1.398 **	
			(0.589)	
PIG-low educated squared			−1.078 **	
			(0.528)	
PIG-high educated				0.867
				(0.550)
PIG-high educated squared				−0.564
				(0.457)
Age	−0.0470 *	−0.0506 *	−0.0525 *	−0.0511 *
	(0.0285)	(0.0278)	(0.0283)	(0.0292)
Age squared	0.000558 **	0.000587 **	0.000618 **	0.000596 **
	(0.000281)	(0.000276)	(0.000280)	(0.000291)
Proportion of working-age members	0.766 ***	0.723 ***	0.801 ***	0.693 ***
	(0.263)	(0.257)	(0.261)	(0.260)
Income	0.0750 *	0.0681 *	0.0814 *	0.0664
	(0.0445)	(0.0413)	(0.0416)	(0.0420)
Education	0.00941	0.00601	0.00768	0.00562
	(0.0146)	(0.0135)	(0.0136)	(0.0139)
Health	0.270 ***	0.265 ***	0.271 ***	0.272 ***
	(0.0679)	(0.0662)	(0.0665)	(0.0679)
Marital status (reference to unmarried)				
Married	−0.118	−0.143	−0.126	−0.144
	(0.277)	(0.276)	(0.279)	(0.278)
Divorced or widowed	−0.252	−0.324	−0.280	−0.298
	(0.307)	(0.305)	(0.308)	(0.309)
Gender(reference to female)	0.0272	0.0112	0.000752	0.0116
	(0.111)	(0.108)	(0.107)	(0.109)
Type of relocation (reference to decentralized resettlement)	0.0155	0.0207	−0.00396	0.00728
	(0.135)	(0.118)	(0.124)	(0.122)
Reason for relocation (reference to poverty alleviation)				
Ecological restoration	0.0496	0.129	0.134	0.131
	(0.191)	(0.187)	(0.190)	(0.193)
Project-induced	−0.310	−0.418 *	−0.348	−0.387 *
	(0.257)	(0.222)	(0.228)	(0.223)
Disaster-related	−0.105	−0.0794	−0.0686	−0.0613
	(0.127)	(0.127)	(0.129)	(0.127)
Other	−0.122	−0.102	−0.0758	−0.0634
	(0.162)	(0.149)	(0.151)	(0.149)
Area (reference to Yan’an)	−0.101	0.00785	−0.0590	0.0190
	(0.212)	(0.188)	(0.199)	(0.189)
cut1	−1.076	−0.861	−0.788	−1.006
	(0.845)	(0.809)	(0.840)	(0.821)
cut2	−0.339	−0.130	−0.0580	−0.278
	(0.842)	(0.806)	(0.837)	(0.819)
cut3	0.533	0.813	0.871	0.657
	(0.843)	(0.809)	(0.840)	(0.821)
cut4	1.846 **	2.153 ***	2.210 ***	1.994 **
	(0.845)	(0.813)	(0.845)	(0.824)
Observations	465	502	496	494

Robust standard errors in parentheses, *** *p* < 0.01, ** *p* < 0.05, * *p* < 0.1.

**Table 4:** Sensitive Groups to Relative Policy Information.

Variables	(10)	(11)	(12)	(13)	(14)	(15)
	SWB	SWB	SWB	SWB	SWB	SWB
PIG	1.950 **	1.550	1.369 *	4.936 **	−1.855	1.973 ***
	(0.882)	(1.269)	(0.768)	(2.260)	(2.578)	(0.757)
PIG squared	−1.535 *	−1.187	−0.891	−4.601 **	2.839	−1.548 **
	(0.843)	(1.173)	(0.721)	(2.155)	(2.419)	(0.719)
Age	−0.0466	−0.0552	−0.0543	0.384	−0.0747	−0.0552 *
	(0.0373)	(0.0437)	(0.0458)	(0.570)	(0.107)	(0.0291)
Age squared	0.000544	0.000740	0.000647	−0.00244	0.000542	0.000641 **
	(0.000359)	(0.000453)	(0.000506)	(0.00379)	(0.00109)	(0.000284)
Proportion of working-age members	0.765 **	0.541	0.485	1.414 **	0.819	0.806 ***
	(0.309)	(0.457)	(0.312)	(0.581)	(1.173)	(0.265)
Income	0.0261	0.144 *	0.0805 *	0.0662	−0.177	0.0596
	(0.0471)	(0.0775)	(0.0434)	(0.129)	(0.163)	(0.0424)
Education	0.00724	0.0168	0.00377	0.00919	0.218 *	0.00264
	(0.0169)	(0.0247)	(0.0141)	(0.0469)	(0.128)	(0.0196)
Health	0.282 ***	0.256 **	0.231 ***	0.480 **	0.348	0.248 ***
	(0.0804)	(0.123)	(0.0722)	(0.190)	(0.299)	(0.0700)
Marital status (reference to unmarried)						
Married	−0.201	0.174	−0.273	1.294	0.442	−0.125
	(0.328)	(0.440)	(0.291)	(1.219)	(1.186)	(0.287)
Divorced or widowed	−0.161	−0.332	−0.701 **	1.725	−1.085	−0.296
	(0.356)	(0.494)	(0.330)	(1.197)	(1.142)	(0.316)
Gender (reference to female)			0.0214	0.307	−1.442 **	0.0342
			(0.117)	(0.331)	(0.570)	(0.112)
Type of relocation (reference to decentralized resettlement)	−0.0453	0.109	0.0174	−0.357	0.323	0.0103
	(0.160)	(0.180)	(0.127)	(0.516)	(0.557)	(0.124)
Reason for relocation (reference to poverty alleviation)						
Ecological restoration	0.246	−0.0767	0.125	0.0699	−1.162	0.234
	(0.286)	(0.257)	(0.208)	(0.397)	(0.835)	(0.201)
Project-induced	−0.309	−0.794 **	−0.432 *	−0.356	−1.306	−0.443 *
	(0.260)	(0.400)	(0.237)	(0.636)	(0.983)	(0.230)
Disaster-related	−0.139	−0.00644	−0.0315	−0.0710	0.336	−0.0963
	(0.161)	(0.216)	(0.140)	(0.315)	(0.694)	(0.133)
Other	−0.316	0.213	−0.0911	0.0840	0.906	−0.166
	(0.198)	(0.237)	(0.163)	(0.491)	(0.752)	(0.150)
Area (reference to Yan’an)	0.0775	−0.463	0.0722	−0.449	−0.375	0.0596
	(0.203)	(0.563)	(0.217)	(0.397)	(0.710)	(0.202)
cut1	−1.120	−0.278	−1.248	17.34	−2.811	−0.875
	(1.005)	(1.409)	(1.035)	(20.76)	(2.632)	(0.873)
cut2	−0.362	0.457	−0.479	18.06	−1.722	−0.149
	(1.010)	(1.387)	(1.027)	(20.75)	(2.571)	(0.868)
cut3	0.606	1.398	0.547	18.66	−0.374	0.786
	(1.013)	(1.387)	(1.027)	(20.74)	(2.621)	(0.871)
cut4	1.897 *	2.878 **	1.929 *	19.96	1.016	2.149 **
	(1.019)	(1.387)	(1.029)	(20.75)	(2.651)	(0.876)
Observations	313	189	429	73	45	457

Robust standard errors in parentheses, *** *p* < 0.01, ** *p* < 0.05, * *p* < 0.1.

**Table 5:** The difference in the impact of PIG on SWB in different policy environments.

Variables	(16)	(17)	(18)	(19)
	SWB	SWB	SWB	SWB
PIG	4.544 ***	0.851	1.761 *	3.300 **
	(1.243)	(0.953)	(0.940)	(1.370)
PIG squared	−3.902 ***	−0.588	−1.335	−2.644 **
	(1.233)	(0.889)	(0.869)	(1.342)
Age	−0.0167	−0.0575 *	−0.0553 *	−0.0600
	(0.0618)	(0.0331)	(0.0324)	(0.0693)
Age squared	0.000320	0.000665 **	0.000617 **	0.000876
	(0.000608)	(0.000320)	(0.000309)	(0.000743)
Proportion of working-age members	−0.0859	1.045 ***	0.925 ***	0.427
	(0.497)	(0.326)	(0.294)	(0.607)
Income	−0.0163	0.112 *	0.0506	0.0394
	(0.0637)	(0.0604)	(0.0484)	(0.0787)
Education	0.000197	0.00971	0.00559	0.0516
	(0.0336)	(0.0232)	(0.0214)	(0.0362)
Health	0.368 ***	0.182 **	0.210 ***	0.456 ***
	(0.142)	(0.0855)	(0.0794)	(0.145)
Marital status (reference to unmarried)				
Married	−0.656	0.00552	−0.0998	−0.131
	(0.702)	(0.347)	(0.358)	(0.416)
Divorced or widowed	−0.848	−0.262	−0.321	−0.253
	(0.745)	(0.389)	(0.398)	(0.477)
Gender (reference to female)	−0.0803	0.000209	0.0222	−0.179
	(0.230)	(0.142)	(0.132)	(0.223)
Type of relocation (reference to decentralized resettlement)	−0.00831	-0.0888		
	(0.228)	(0.164)		
Reason for relocation (reference to poverty alleviation)				
Ecological restoration	0.244	0.324	0.316	−0.575 *
	(0.370)	(0.236)	(0.220)	(0.311)
Project-induced	−0.515 *	−0.667 *	−0.308	−0.945 **
	(0.304)	(0.398)	(0.273)	(0.435)
Disaster-related	0.0487	−0.168	−0.0439	−0.417
	(0.277)	(0.158)	(0.149)	(0.294)
Other	−0.161	−0.116	−0.225	−0.264
	(0.288)	(0.176)	(0.184)	(0.266)
Area(reference to Yan’an)	−0.01000	−0.00538	0.0366	0.201
	(0.376)	(0.224)	(0.236)	(0.348)
cut1	−1.314	-0.574	−1.023	−0.179
	(1.573)	(1.082)	(1.015)	(1.688)
cut2	−0.306	0.0745	−0.320	0.590
	(1.527)	(1.082)	(1.015)	(1.656)
cut3	0.577	1.010	0.641	1.495
	(1.521)	(1.088)	(1.021)	(1.648)
cut4	1.913	2.430 **	1.979 *	2.860 *
	(1.515)	(1.099)	(1.027)	(1.638)
Observations	137	301	353	124

Robust standard errors in parentheses, *** *p* < 0.01, ** *p* < 0.05, * *p* < 0.1.
